# Micrococcal Nuclease stimulates *Staphylococcus aureus* Biofilm Formation in a Murine Implant Infection Model

**DOI:** 10.3389/fcimb.2021.799845

**Published:** 2022-01-17

**Authors:** Abigail M. Forson, Colin W. K. Rosman, Theo G. van Kooten, Henny C. van der Mei, Jelmer Sjollema

**Affiliations:** University Medical Center Groningen, Department of Biomedical Engineering, University of Groningen, Groningen, Netherlands

**Keywords:** biofilm, neutrophil extracellular trap, micrococcal nuclease, immune evasion, *Staphylococcus aureus*, biomaterial associated infection, mesh implant

## Abstract

Advancements in contemporary medicine have led to an increasing life expectancy which has broadened the application of biomaterial implants. As each implant procedure has an innate risk of infection, the number of biomaterial-associated infections keeps rising. *Staphylococcus aureus* causes 34% of such infections and is known as a potent biofilm producer. By secreting micrococcal nuclease *S. aureus* is able to escape neutrophil extracellular traps by cleaving their DNA-backbone. Also, micrococcal nuclease potentially limits biofilm growth and adhesion by cleaving extracellular DNA, an important constituent of biofilms. This study aimed to evaluate the impact of micrococcal nuclease on infection persistence and biofilm formation in a murine biomaterial-associated infection-model with polyvinylidene-fluoride mesh implants inoculated with bioluminescent *S*. *aureus* or its isogenic micrococcal nuclease deficient mutant. Supported by results based on *in-vivo* bioluminescence imaging, *ex-vivo* colony forming unit counts, and histological analysis it was found that production of micrococcal nuclease enables *S. aureus* bacteria to evade the immune response around an implant resulting in a persistent infection. As a novel finding, histological analysis provided clear indications that the production of micrococcal nuclease stimulates *S. aureus* to form biofilms, the presence of which extended neutrophil extracellular trap formation up to 13 days after mesh implantation. Since micrococcal nuclease production appeared vital for the persistence of *S. aureus* biomaterial-associated infection, targeting its production could be a novel strategy in preventing biomaterial-associated infection.

## 1 Introduction

Infection is a dreaded but common complication after implant surgery. Often, long antibiotic treatments and multiple surgeries are required to treat the infection and replace the implant, resulting in prolonged hospitalization (Major et al., 2015; Sjollema et al., 2018). Advancements in contemporary medicine, have led to an increase in life expectancy and an aged population which has in turn broadened the application of biomaterial implants (Major et al., 2015). For example, biomaterial implants are utilized in hernia repair, joint arthroplasty, replacement of cardiac valves, vascular replacement and bypass surgery among others. Each implant procedure has an innate risk of causing an infection. Therefore, increased use of biomaterials is accompanied by a high incidence of biomaterial-associated infections (Sjollema et al., 2018) *Staphylococcus aureus* is the cause of 34% of all biomaterial-associated infections (Arciola et al., 2005) and is known as a strong biofilm producer. Biofilms are organized aggregates of bacteria enclosed in a self-produced protective matrix of extracellular polymeric substances (EPS). The main constituents of the EPS matrix are polysaccharides, proteins and extracellular DNA (eDNA) which in particular has been shown to hold the biofilm structure together through interactions with resident bacteria and proteins (Mann et al., 2009; Dengler et al., 2015). Biofilms protect bacteria by shielding them from external factors like the host-immune system and antibiotics (Zimmerli and Sendi, 2011). Biofilms also serve as a source for bacterial dissemination to the entire body (Zimmerli and Sendi, 2011).

Biomaterial implants are not biologically inert and elicit an immune response from the host, often referred to as the foreign body reaction. The foreign body reaction is a process where an implant evokes an inflammatory reaction which recruits neutrophils, macrophages, and other immunomodulatory cells to the implant site often resulting in the enclosure of the biomaterial in fibrous tissue (Anderson et al., 2008). The early stages of the foreign body reaction are marked by the transient presence of neutrophils and extensive production of neutrophil extracellular traps by activated neutrophils around these biomaterial implants (Jhunjhunwala et al., 2015; Vitkov et al., 2015; Oe et al., 2021). Neutrophil extracellular traps are composed of DNA fibers and antimicrobial peptides such as citrullinated histones, myeloperoxidase, neutrophil elastase and cathepsin (Brinkmann et al., 2004; Wang et al., 2009). Neutrophils can interact with the biofilm EPS matrix and produce neutrophil extracellular traps in an attempt to clear infections (Hirschfeld, 2014). *In vitro* studies have shown that neutrophils can even enter *S. aureus* biofilms and release neutrophil extracellular traps. Also, they may phagocytose sections of *S. aureus* biofilms without successfully eliminating the whole biofilm (Günther et al., 2009; Bhattacharya et al., 2018).

Similarly, production of neutrophil extracellular traps can be induced by the interaction of neutrophils with protein A on the surface of *S. aureus* (Hoppenbrouwers et al., 2018), serum platelets (Tanner et al., 2021), and biofilm leukocidins (Lei et al., 2017; Bhattacharya et al., 2018). Neutrophil extracellular traps can trap bacteria, and besides killing the bacteria with the aforementioned antimicrobial peptides, the phosphodiester backbone of neutrophil extracellular traps DNA can cause contact-dependent lysis of bacteria by chelating cations that stabilize the bacterial cell surface (Brinkmann et al., 2004; Wang et al., 2009; Halverson et al., 2015). However*, S. aureus* is able to escape neutrophil extracellular traps by secreting micrococcal nuclease (Brinkmann et al., 2004), an endonuclease with the ability to cleave DNA into mono- and dinucleotides, thereby destroying the DNA scaffold of the neutrophil extracellular traps (Tang et al., 2011; Thammavongsa et al., 2013; Kiedrowski et al., 2014). The secreted micrococcal nuclease (termed Nuc1) has a higher expression and nuclease activity than the membrane-bound micrococcal nuclease (termed Nuc2) (Hu et al., 2012; Kiedrowski et al., 2014). In addition to its role in escaping from neutrophil extracellular traps, micrococcal nuclease also facilitates biofilm dispersal by separating small clusters of bacteria by cleaving biofilm eDNA (Moormeier et al., 2014). Several studies have reported that micrococcal nuclease limits biofilm growth and adhesion, but results are variable depending on strain and experimental conditions (Kiedrowski et al., 2011; Tang et al., 2011; Beenken et al., 2012).

In the case of a biomaterial-associated infection, it is expected that a strong immune response, accompanied by a high production of neutrophil extracellular traps, is induced. Bacteria may react by increasing the production of nucleases to degrade neutrophil extracellular traps, thereby jeopardizing the integrity of the protective EPS matrix. Whether nuclease activity, resulting in eDNA cleavage and degradation of neutrophil extracellular traps, promotes bacteria in the planktonic state rather than in a biofilm mode is still unclear. Therefore, this study aimed at identifying the impact of micrococcal nuclease on both infection severity and persistence and on biofilm formation. To this end we applied a murine subcutaneous implant infection model with an implanted polyvinylidene fluoride (PVDF) mesh infected with bioluminescent *S. aureus* Newman or its isogenic *Δnuc1* mutant (Kiedrowski et al., 2011; Hernandez et al., 2014). The PVDF mesh has been characterized as biocompatible and is commonly used in surgical procedures in abdominal wall hernia repair (Klinge et al., 2002).

## 2 Materials and Methods

### 2.1 Bacterial Strains and Cultures

All media were prepared according to manufacturer’s protocol. *S. aureus* Newman strains were kindly donated by Prof. McNamara (Department of Internal Medicine, University of Illinois, USA). Bioluminiscent *S. aureus* Newman WT lux (Hernandez et al., 2014) and *S. aureus* Newman *Δnuc1* lux (Kiedrowski et al., 2011; Hernandez et al., 2014) were cultured from cryopreservative beads onto Tryptane Soy Agar (TSA) (Oxoid Ltd., Basingstoke, UK). 200 μg/mL kanamycin was added to the agar plates, pre-, and main-cultures of the *S. aureus* Newman lux strains. After inoculating the agar plates with the beads, the plates were incubated for 24 h at 37°C in ambient air. *S. aureus* Newman is a MSSA strain, isolated from a case of tubercular osteomyelitis in a patient (Duthie and Lorenz, 2018). The *S. aureus* Newman *Δnuc1* was constructed by employing the Targetron Gene Knockout system (Kiedrowski et al., 2011). This mutation resulted in loss of nuclease activity in *Δnuc1* mutant strain and nuclease activity was restored with complementation of *nuc1* on a plasmid. Subsequently bacteriophage φ11 was used to transduce the Photorhabdus luminescens derived luciferase and kanamycin resistance genes luxABCDE-Kan from the *S. aureus* Xen29 (AH1362) to the *S. aureus* Newman and *Δnuc1* mutant strains (Novick, 1991; Hernandez et al., 2014). Both the nuclease deficient as the WT strain were shown to produce equal amounts of bioluminescence radiance (Hernandez et al., 2014).

Prior to each experiment, bioluminescence of the colonies on the agar plate was confirmed and a pre-culture was made from such a colony. With this colony, 10 mL of tryptone soy broth (TSB, Oxoid Ltd.) was inoculated and cultured for 24 h at 37°C, 150 RPM. The main culture was made by inoculating 40 mL TSB with 2 mL of the pre-culture and grown for 16 h at 37°C,150 RPM. The main culture was centrifuged for 5 min at 10°C, 5000 g and subsequently washed three times with phosphate buffered saline (PBS, 10 mM potassium phosphate, 150 mM NaCl, pH 7.4). Next, all cultures were sonicated 3 times for 10 s at 30 Watts. The number of bacteria was counted in a Bürker-Türk counting chamber, and bacteria were resuspended in PBS at appropriate concentrations.

### 2.2 Animals

Female Balb/c OlaHsd mice of 4-5 weeks old and an average weight of 20 g were used. Animals were examined every two days, noting aberrations in behaviour such as apathy, appearance of the skin, weight loss, and signs of local infection such as pus secretion and subcutaneous swelling/abcesses. Humane endpoints were defined in advance and included: visual indications for apathy, weight loss >15% of the starting weight, excessive pus secretion, redness and/or swelling persisting for more than 4 days, and mesh implants penetrating the skin. Upon meeting any humane endpoint animals were terminated.

Animals were anesthesised with 2% isoflurane gas during every procedure descibed below. At T=0 days the skin was shaved and desinfected with chlorhexidine. A 1 cm transverse transcutaneous incision was made in the dorsal cervical area. Using blunt dissection, a subcutaneous pocket was created in the caudal direction. When indicated, a sterile 1 cm^2^ PVDF mesh (Dynamesh, FeG, Germany) was inserted. The wounds were closed using Histoacryl (Braun, Germany), after which an injection with 20 µL sterile PBS or bacterial inoculum was administered in the pocket. Finally, 0.05 mg/kg Temgesic (Indivior UK Limited, United Kingdom) was administered subcutaneously for pain relief. When indicated, mice were terminated using cervical dislocation after retrieving the tissue samples under aneasthesia.

### 2.3 Dose Finding and Main Study Setup

To determine the optimal dose for infection of the animal model, a dose-finding study was performed. Six groups of mice (n=4) received an implant and were infected with 10^6^, 10^7^, or 10^8^ bacteria of either the *S. aureus* Newman WT lux or the *S. aureus* Newman *Δnuc1* lux in 20 µL of PBS. Another six groups (n=2) received a sham surgery and were similarly injected with one of the strains, at the aforementioned doses. Infection progression was measured by bioluminescent imaging at T=0, 2, 4, 6, and 10 days as described in section *Bioluminescence Imaging*. Based on the results of the dose-finding study (**Figure S1**) the inoculum in the main study was set at 10^8^ bacteria since at this concentration measured bioluminescence in mice with an implant was more persistant than in mice without an implant, which is generally considered to be characteristic for biomaterial-associated infections.

Since the primary endpoint of our research was the influence of Nuc1 on the *S. aureus* biofilm in *in vivo* implant infections, we based our power analysis for determining the number of mice in the main experiment on bioluminescence measurements obtained previously. The average bioluminescence signal of an *S. aureus* mesh infection, taking into account repetitive measurments in one and the same mouse, was 4x10^5^ ± 1.5x10^5^ p/s (Daghighi et al., 2015). Furthermore, we estimated an expected effect size of 2x10^5^ p/s, and executed the power analysis with a power of 0.8 and alpha of 5%. Taking into account 10% implant loss, the final group size was 10 mice in both arms of the study (*S. aureus* Newman WT lux or the *S. aureus* Newman *Δnuc1* lux). The number of mice in the control group was lower (n=4) because of the lack of an infection and lower number of degrees of freedom. Mice in the main study were divided into 11 groups (**Table S1**). Two groups of mice (n=7), to be sacrificed at T=7, received an implant and were inoculated with 10^8^ bacteria of either the *S. aureus* Newman WT lux or the *S. aureus* Newman *Δnuc1* lux. Two more groups were prepapred in the same way, to be sacrificed at T=13 (n=3). Mice in the two control groups underwent the same surgical procedure, but were injected with sterile PBS and terminated at T=7 (n=4) or T=13 (n=3).

Four groups of mice (n=3) received a sham surgery and were similarly injected with one of the bacterial strains at the aforementioned dose and designated for sacrifice at either T=7 or T=13. Two groups of mice received an implant and were injected with sterile PBS (n=4) or received no implant and PBS (n=3). Mice in the control group underwent the same procedure and were injected with sterile PBS and sacrificed at T=7 (n=3). No control group was added for sham surgery, sterile PBS injection, and sacrifice at T=13. This would not provide new information compared to the group sacrificed at T=7 and therefore not be ethically justifiable.

Up to day 7, there was a total of 10 mice in each group with an implant and an *S. aureus* infection available for bioluminescent imaging. 7 of the 10 mice were sacrificed at T= 7 and 3 mice experienced an extended course of infection up to T=13. Mice infected with *S. aureus* Newman WT lux were never kept in the same cage as mice infected with *S. aureus* Newman *Δnuc1* lux.

Bioluminescence imaging of the 7-day group took place at T=0, 1, 3, 5, 7 days. Imaging of the 13-day group took place at T=0, 3, 7, 10, 13 days. At T= 12 days two animals were at risk of implant loss, as a single mesh fibre was penetrating through the skin. These animals were terminated after imaging the same day and processed as described in section *Bioluminescence Imaging, Ex-vivo Examination, CFU Counting*.

### 2.4 Bioluminescence Imaging

Bioluminescence was measured using the IVIS spectrum system (PerkinElmer, Waltham Massachusetts, USA). Data was analyzed using the LivingImage 4.7.2 software (PerkinElmer). All animals were sedated using isoflurane anesthesia during imaging. When measuring bioluminescence the imaging time was automatically set by the mentioned software based on the actual luminescence in order to prevent under- and oversaturation. Emission filter was set to “open”, excitation filter to “block” and binning to factor 8. All animals were imaged using the same field of view. Positioning of the region of interest was at the site of the subcutaneous mesh and done using the histoacryl glue (which was visible during the entire experiment) as a landmark. The area of the region of interest was identical in all animals (21.2 mm^2^). Background luminescence was calculated from an empty part of the image and subtracted from the luminescence from the regions of interest.

### 2.5 *Ex Vivo* Examination

To retrieve tissue samples the dorsal side of the mice was sanitised with chlorhexidine in 70% ethanol and the implant was excised, including the adherent skin and any fibrous material, excluding muscle tissue. When a mouse did not receive an implant a section of tissue of equal size around the injection site (1x1x0.2 cm) was taken. Half of this sample was used for CFU counting, and half for histological analysis.

### 2.6 CFU Counting

Samples were stored in sterile eppendorf tubes on ice, containing glass beads (1 mm) and 1 mL of Reduced Transport Fluid, a medium to preserve sample integrity (Syed and Loesche, 1972). Samples were subsequently homogenized by the bead-beater (3000 bpm, 1 min), 30 s of vortexing, 5 min of sonification in a sonification bath, and another 30 s of vortexing. Then samples were serially diluted 1:10 and three 10 µL drops of the first seven dilutions (10^0^-10^7^ times diluted) were plated on TSA. After 16 h of incubation at 37°C all plates were imaged in the IVIS to count the number of luminescent colonies. Only luminescent colonies were included in the analysis, as non-luminescent colonies were regarded as contaminations.

### 2.7 Histological Analysis

Samples were fixed overnight in 4% paraformaldehyde at 4°C and dehydrated by incubating in ascending concentrations of ethanol (70/80/90/100%), followed by washing with xylene, before paraffin embedding. Slices of 5 μm thickness were cut from the paraffin embedded samples with a microtome. The slices were deparaffinized by incubating in two changes of xylene (EMSURE, Darmstadt, Germany) for 5 min each. This step was followed by rehydration of the samples by immersion in decreasing concentrations of ethanol (100-96-70% ethanol) and finally washed 3 times in demineralized water. Slices were stained with Mayer’s hematoxylin (Sigma, St. Louis, USA) for 5 min, followed by several washing steps in demineralized water to remove excess hematoxylin. Bluing of hematoxylin was done by incubating slices in tap water for 5 min. Hereafter, staining with Eosin (Eosin Y, Sigma) was performed for 40 s. This was followed by washing 3 times in 96% ethanol for 5 min each. Slices were dehydrated by incubating in 100% ethanol for 15 min, followed by a 45 min incubation in xylene. The stained slices were covered with permanent mounting medium (Permount, Fisher Scientific, New Jersey, USA) and then with glass cover slips. Images were obtained with a light microscope after drying overnight.

### 2.8 Immunohistochemistry

For immunodetection of bacteria and neutrophil extracellular traps, the following antibodies were used: polyclonal Rabbit anti *S. aureus* antibody (Bio-Rad, Hercules, California, USA), Rabbit polyclonal to myeloperoxidase (ab9535, Abcam, Cambridge, United Kingdom), Rabbit polyclonal to Histone H3 (citrulline R2+R17) (ab5103, Abcam) and polyclonal Goat Anti-Rabbit immunoglobulin biotinylated (Dako, Santa Clara, CA, USA). All immunohistochemical staining were performed on slices which were deparaffinized and rehydrated as described above. The slices were subjected to heat induced epitope retrieval to unmask antibody binding sites in citrate buffer (10 mM citric acid, 0.05% Tween20, pH 6). After this, the samples were washed twice with PBS supplemented with 0.05% Tween20. Blocking of endogenous binding sites was performed by incubating the sections in PBS supplemented with 5% goat serum (Bio-Rad). Subsequently, slices were incubated with primary antibody: Rabbit anti *S. aureus* antibody (1:50), Rabbit polyclonal to myeloperoxidase antibody (1:100) or Rabbit polyclonal to Histone H3 (citrulline R2+R17) (1:50) for 20 h at 4°C to target *S. aureus* protein A, myeloperoxidase and citrullinated histones, respectively. As a negative control for each antibody staining, primary antibody was replaced with equal amount of antibody diluent (PBS supplemented with 1% goat serum). After washing with PBS, the slices were treated with 0.3% H_2_O_2_ for 10 min in darkness to quench endogenous peroxidase activity. After washing with PBS, bound antibodies were detected with polyclonal, biotinylated Goat Anti-Rabbit immunoglobulin (1:2000) secondary antibodies for 45 min at room temperature. After washing with PBS, slices were treated with Avidin-Biotinylated Horseradish Peroxidase complex (ABC Peroxidase staining kit, Thermo scientific, Illinois, USA) according to the manufacturer’s instructions. Sections were then stained with the substrate 3,3’-diaminobenzidine tetrahydrochloride (DAB, Thermo Scientific) and counterstained with Mayer’s hematoxylin for 1 min. After being oxidized by peroxidase, DAB precipitated with a dark brown color. Excess hematoxylin was removed by washing the samples in demineralized water for 30 s. Bluing of the hematoxylin stain was done by a 5 min incubation in tap water. This step was followed by ethanol dehydration in increasing concentrations of ethanol (70/96/100%) and a 6 min incubation in xylene. The samples were covered with permanent mounting medium and then glass cover slips and allowed to dry overnight. Images were acquired with a light microscope.

Unless only one filament was found, four randomly selected images of mesh filaments, at 400x magnification were utilized to semi-quantitatively analyze the presence of bacteria and components of neutrophil extracellular traps by determining the mean grey value of the brown channel after color deconvolution with ImageJ, following an integrated protocol (Crowe and Yue, 2019).

### 2.9 Statistical Analysis

Statistical analysis was performed using Graphpad Prism 8.0.1 (Graphpad, San Diego, United States). Unless stated differently, statistical differences were calculated using one-way ANOVA, correcting for multiple testing where indicated. Differences were considered statistically significant when P < 0.05.

## 3 Results

### 3.1 Animal Survival

All mice survived during the experiment after the implantation of a sterile implant. Humane endpoints (excessive swelling/pus or loss of implant) were reached in 4 mice with both a bacterial infection and an implant at T=9 (1 mouse), T=10 (1 mouse), and T=12 days (2 mice) (**Figure S2**). One mouse with an implant inoculated with *S. aureus* Newman WT lux died unexpectedly after 5 days, showing no suspect signs at all. Nuclease activity did not significantly impact animal survivability.

### 3.2 Bioluminescence of the Bacterial Infection in Mice

**Figure 1:**
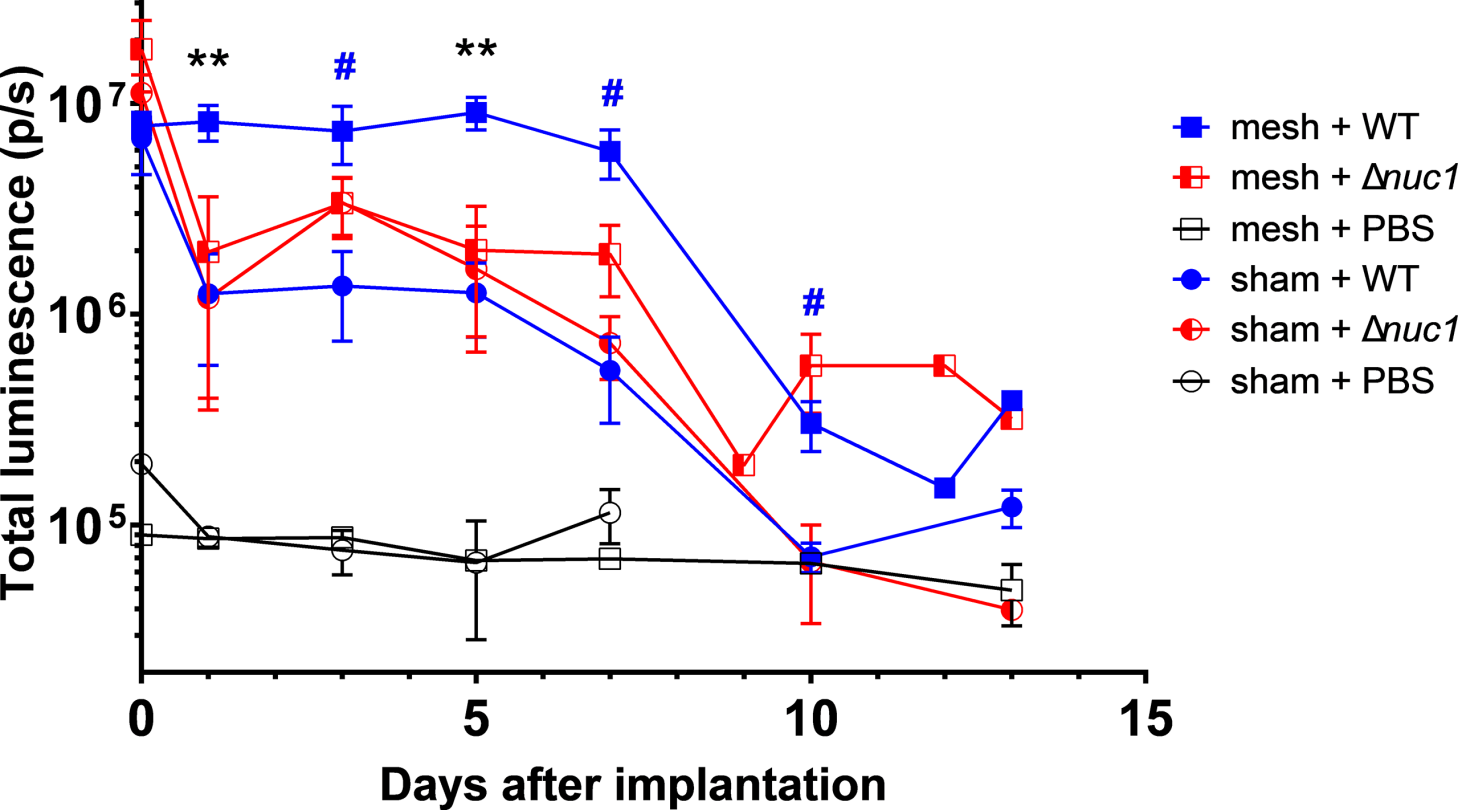
Bacterial bioluminescence measured during the course of infection in PVDF surgical mesh implanted mice inoculated with either *S. aureus* Newman WT lux (WT), *S. aureus* Newman *Δnuc1* lux (Δ*nuc1*), or sterile PBS. Sham indicates mice that underwent surgery without implantation of a mesh. Error bars represent standard error of the mean. Differences were analyzed using a repeated measures ANOVA, corrected for multiple measuring and lognormal values. Lognormality was confirmed using a Q-Q plot. Black asterisks indicate significant differences between mesh + WT and mesh + Δnuc1, blue hashtags indicate significant difference between mesh + WT and sham + WT. ^#^P ≤ 0.05; **P ≤ 0.01. Numbers of mice included in the statistical analysis.

From day 1 up to day 7, the bioluminescence measured in mice with a mesh implant inoculated with *S. aureus* Newman WT lux, was significantly higher than in mice that received a mesh implant inoculated with *S. aureus* Newman Δ*nuc1* lux and higher than mice inoculated with bacteria but lacking an implant (**Figures 1**, **S3**). A significant 3- to 10-fold decrease in bioluminescence was observed at day 1 in all groups except in the group of mice with an implant and inoculated with *S. aureus* Newman WT lux, which remained high. From day 1 mice infected with *S. aureus* Newman Δ*nuc1* lux showed equal bioluminescence, irrespective of implant presence. Further reduction in bioluminescence was observed in all groups infected with bacteria between day 7 and 10.

In the case of mice that did not obtain a bacterial inoculation, the bioluminescent signal is stable around 8x10^4^ p/s. This bioluminescence signal is at the level of the background noise of the imaging system that is basically around 10^5^ p/s. Background signal or noise is usually generated as temperature induced dark current and read out noise of the CCD chip (Sjollema et al., 2010). It should also be noted that at the day of inoculation both strains induced similar bioluminescence radiance, which again confirmed that both strains produced equal amounts of bioluminescence.

### 3.3 CFUs of All Groups at Termination

**Figure 2:**
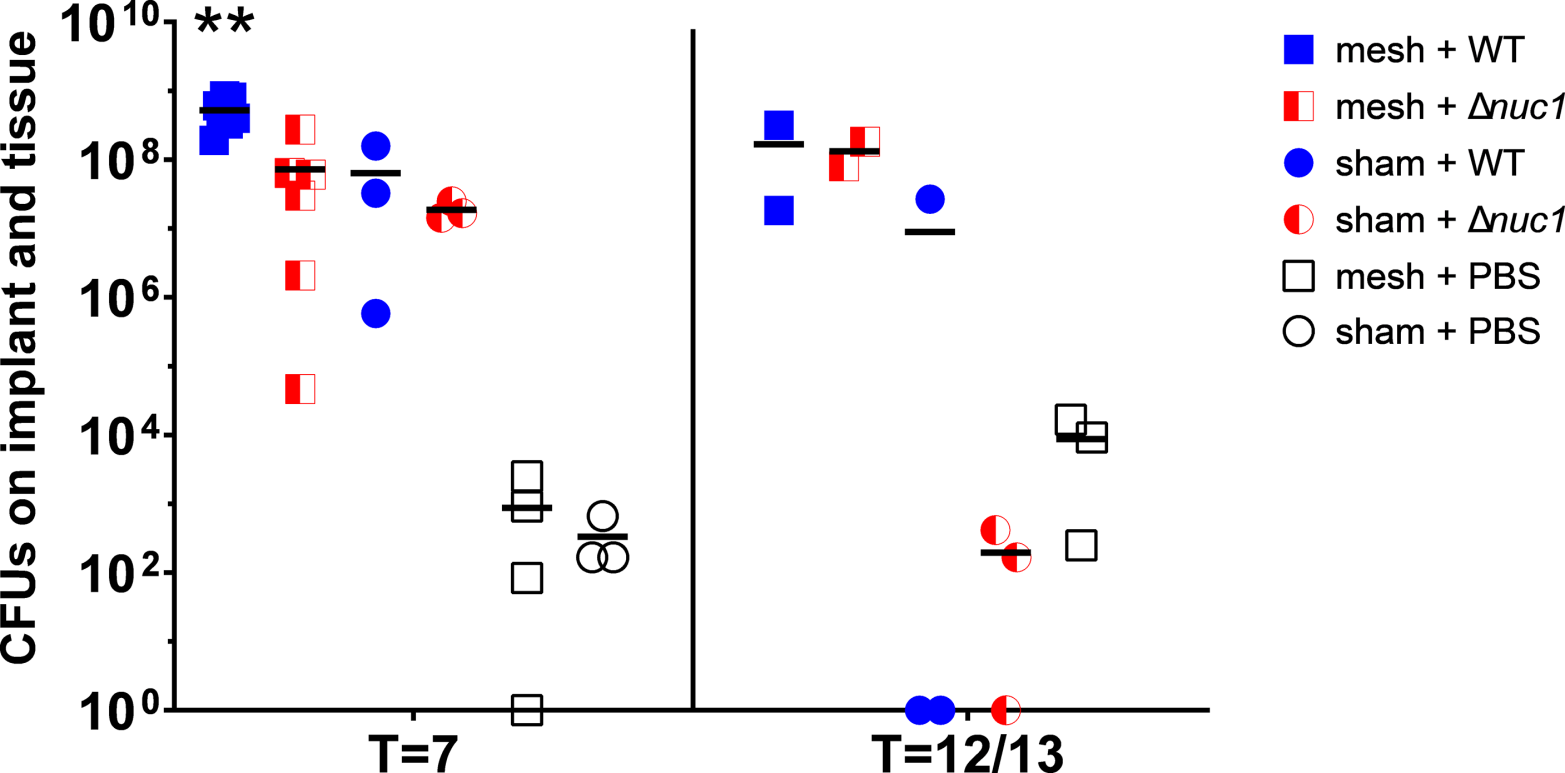
Total CFUs in mesh-tissue samples taken after termination of mice inoculated with either *S. aureus* Newman WT lux (WT) or *S. aureus* Newman *Δnuc1* lux (*Δnuc1*) strains or injected with sterile PBS after 7 days or after 12 or 13 days post implantation (two measurements were taken at T=12, and the other at T=13 days) of PVDF surgical mesh implant. Horizontal bar represents the geometric mean value. **Indicates significant difference between mesh + WT and all other groups at the same timepoint (P < 0.01).

At 7 days after implantation, significantly more CFUs were found in the samples from mice with an implant inoculated with *S. aureus* Newman WT lux compared to mice with an implant that were inoculated with *S. aureus* Newman Δ*nuc1* lux (P=0.0013), or compared to mice that did not receive an implant at all and were inoculated with either *S. aureus* Newman Δ*nuc1* lux group (P=0.003) or WT (P=0.007) (**Figure 2**). This is analogous to the results shown by measuring luminescence. Note that tissue from mice inoculated with sterile PBS contained also some bacteria, this contamination was probably caused during the explantation process. Such small contaminations could not have an effect on the analysis of the infected groups since the number of CFU’s in these groups were 10^4^-10^5^ times higher. A small number of colonies on the agar plates obtained from biopsies from the infected mice have been tested for nuclease production and no WT bacteria were found in the group infected with the Δ*nuc1* mutant strains or vice versa (data not shown), which indicates that no cross contaminations took place during implantation and inoculation. Nuclease production was assessed by a nuclease activatable fluorescence probe (Rosman et al., 2018).

At T=12 or 13 days (two measurements were taken at T=12, and the other at T=13 days*)* no significant differences were observed between any groups.

### 3.4 Tissue Response

**Figure 3:**
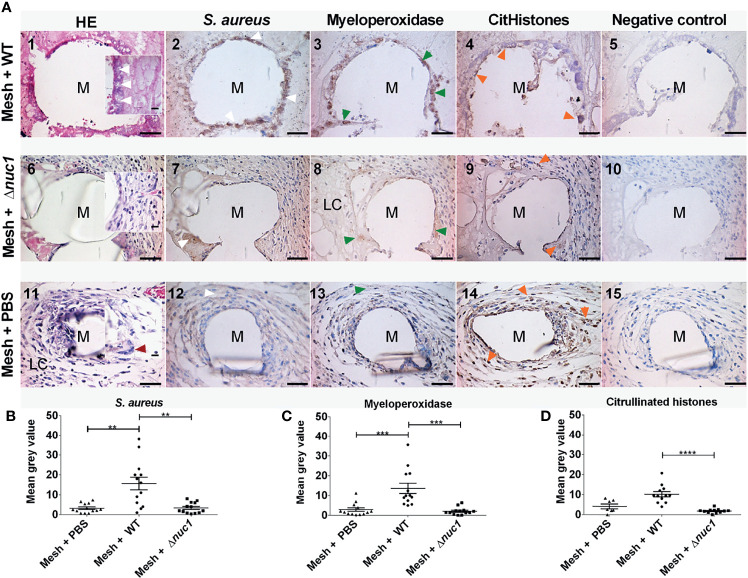
**(A)** Representative images of hematoxylin (dark blue) and eosin (pink) staining and immune-histochemical detection (brown staining) of *S. aureus*, myeloperoxidase and citrullinated histones in mice tissue biopsies at positions surrounding a single filament of a PVDF surgical mesh implant (M) in combination with an *S. aureus* Newman WT lux, *S. aureus* Newman *Δnuc1* lux or sterile PBS injection, 7 days after implantation. Each image in a row corresponds to the same location in the sample taken from one mouse. White arrowheads show locations of bacterial biofilms, dark red arrowheads show locations of foreign body giant cells, green arrowheads show locations of myeloperoxidase and orange arrowheads show locations of citrullinated histones. As a negative control for each antibody staining, primary antibody was replaced with equal amounts of antibody diluent. **(B)** Semi-quantitative analysis of immunohistochemically determined *S. aureus*, **(C)** myeloperoxidase and **(D)** citrullinated histones. Data points represent data from tissue from 3 different mice and 4 filaments from each mouse were analyzed except when only one filament was present, N=12. Error bars show the standard error of the mean. Statistical significance between mice with different infection is indicated with asterisks, **P ≤ 0.01, ***P ≤ 0.001, ****P ≤ 0.0001. Scale bar= 50 µm, scale bar insert=25 µm.

**Figure 4:**
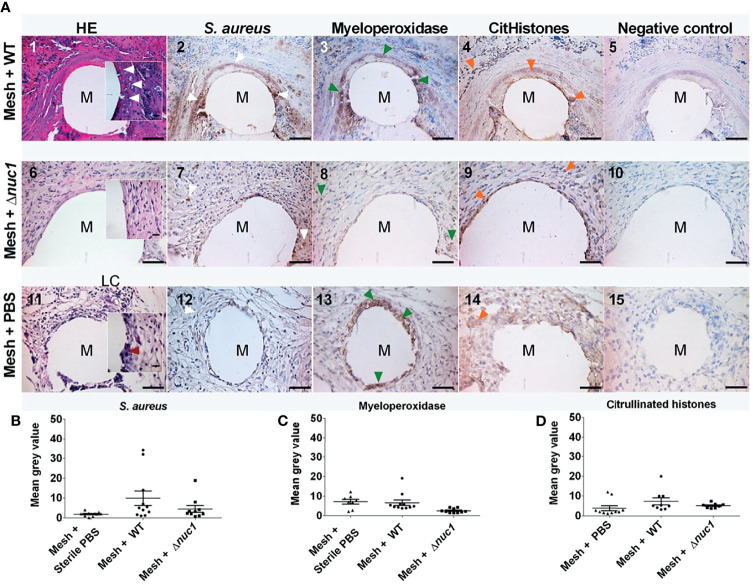
**(A)** Representative images of hematoxylin (dark blue) and eosin (pink) staining and immune-histochemical detection (brown staining) hematoxylin of *S. aureus*, myeloperoxidase and citrullinated histones in mice tissue biopsies at positions surrounding a single filament of a PVDF surgical mesh implant (M) in combination with an *S. aureus* WT lux, *S. aureus* Newman *Δnuc1* lux or sterile PBS injection. Biopsies were taken from mice terminated 12 and 13 days after implantation. Each image in a row corresponds to the same location in the sample taken from one mouse. White arrowheads show locations of bacterial biofilms, dark red arrowheads show locations of foreign body giant cells, green arrowheads show locations of myeloperoxidase and orange arrowheads show locations of citrullinated histones (CitHistones). As a negative control for each antibody staining, primary antibody was replaced with equal amounts of antibody diluent. **(B)** Semi-quantitative analysis of immunohistochemically determined *S. aureus*, **(C)**, myeloperoxidase and **(D)** citrullinated histones. Data points represent data from tissue from 2 different mice per group of infected mice and 3 in the group of sterile mice and 4 filaments from each mouse were analyzed except when only 1 filament was present in a single biopsy. Error bars show the standard error of the mean.

Tissue histology was examined after hematoxylin and eosin staining in *ex-vivo* samples taken 7 or 13 days after implantation of the mesh (images 1, 6 and 11 of **Figures 3A**, **4A**, respectively). The filaments of the mesh appeared as disc-shaped openings with a diameter of 144 ± 18 µm, falling within the 85-165 µm diameter range of the PVDF filaments. At day 7, the pink eosin staining showed the presence of proteins in the mice that received a mesh and were inoculated with *S. aureus* Newman WT lux. Bacteria appeared as purple granules at the mesh-tissue interface (**Figure 3A-1** insert, white arrowheads). The samples, however, lacked the characteristic deep blue nuclei stain from hematoxylin, indicating that the area around the filaments were depleted of immune cells. At day 13, the tissue surrounding the filaments was dense and heavily stained by eosin as was the case at day 7. However, immune cells with deep blue, hematoxylin-stained nuclei had moved closer to the filaments. Bacteria were still visible as purple granules at the mesh-tissue interface (white arrowheads, **Figure 4A-1**)

In contrast, tissue cells had gained access to the PVDF mesh filament in the group of mice that received a mesh and were inoculated with *S. aureus* Newman Δ*nuc1* lux at day 7. Tissue surrounding the mesh filaments showed an organized granulation tissue where a single layer of cells was found at the filament-tissue interface, followed by cells with round and ellipsoid nuclei which were identified as immune and fibroblast-like cells, respectively (**Figure 3A-6**). Other sections of the tissue surrounding the mesh filaments consisted of loose connective tissue (LC, **Figure 3A-8**). At day 13, loose connective tissue that surrounded the mesh filaments at day 7 had been replaced with a denser tissue with immune cells and fibroblast-like cells (**Figure 4A-6**). Also, foreign body giant cells were identified in the vicinity of the mesh filaments (**Table 1**). In the group inoculated with PBS, the tissue surrounding the mesh filaments consisted of loose connective tissue with immune cells and fibroblast-like cells as well as foreign body giant cells (LC, red arrowhead, **Figure 3A-11**). Foreign body giant cells were not found in samples obtained from mice that received an implant and were inoculated with *S. aureus* Newman WT lux (**Table 1**). At day 13, a layer of cells lined the mesh-tissue interface with foreign body giant cells in close proximity to the mesh. This was followed by loose connective tissue, scattered with immune cells and fibroblast-like cells (**Figure 4A-11**).

**Table 1:** Appearance of foreign body giant cells (FBGCs) in the vicinity of the PVDF surgical mesh implant mesh implant.

Group	Days afterimplantation	Number of filaments with FBGCs	Total number of examined filaments
PVDF surgical mesh implant *+* *S. aureus* Newman WT lux	7	0	31
PVDF surgical mesh implant + *S. aureus* Δnuc1 lux	7	0	23
PVDF surgical mesh implant + PBS	7	3	15
PVDF surgical mesh implant + *S. aureus* Newman WT lux	12 or 13	0	18
PVDF surgical mesh implant + *S. aureus* Δnuc1 lux	12 or 13	6	13
PVDF surgical mesh implant + PBS	13	15	24

### 3.5 Immunohistochemical Analysis

#### 3.5.1 *S. aureus* Detection

Antibodies against the *S. aureus* surface protein A were utilized to detect bacteria in *ex-vivo* samples taken 7 and 13 days after implantation of the mesh in mice that received a *S. aureus* Newman WT lux or *S. aureus* Newman Δ*nuc1* lux bacteria or sterile PBS. Bacterial biofilms (white arrows) were found both at the interface of the mesh filaments as well as at positions located deeper in the tissue (**Figures 3A-2**, **4A-2**). The protein A antibody staining colocalized with an alcian blue staining of polysaccharides, which confirms that bacteria around the filaments indeed participate in a biofilm (**Figure S4**. This staining procedure has been utilized earlier to detect biofilms in clinical samples (Wu et al., 2020). Semi quantitative analysis of protein A staining showed that at day 7, the presence of *S. aureus* Newman WT lux as significantly higher (P ≤ 0.01) than that of *S. aureus* Newman *nuc1* lux as well as the group inoculated with PBS (P ≤ 0.01) (**Figure 3B**). *S. aureus* Newman WT lux appeared well detectable in samples taken 13 days after implantation, though not in significantly higher amounts than *S. aureus* Newman Δ*nuc1* lux or the group inoculated with PBS (**Figure 4B**). Negative controls with only secondary antibody did not show the characteristic brown DAB precipitate (images 5, 10, 15 of **Figures 3A**, **4A**). Statistical analysis performed for the Δ*nuc1* group at 13 days is based on data from two out of the three tissues that were selected for histological analysis since the third sample had no mesh filaments.

#### 3.5.2 Detection of Neutrophil Extracellular Trap Components

**Figure 5:**
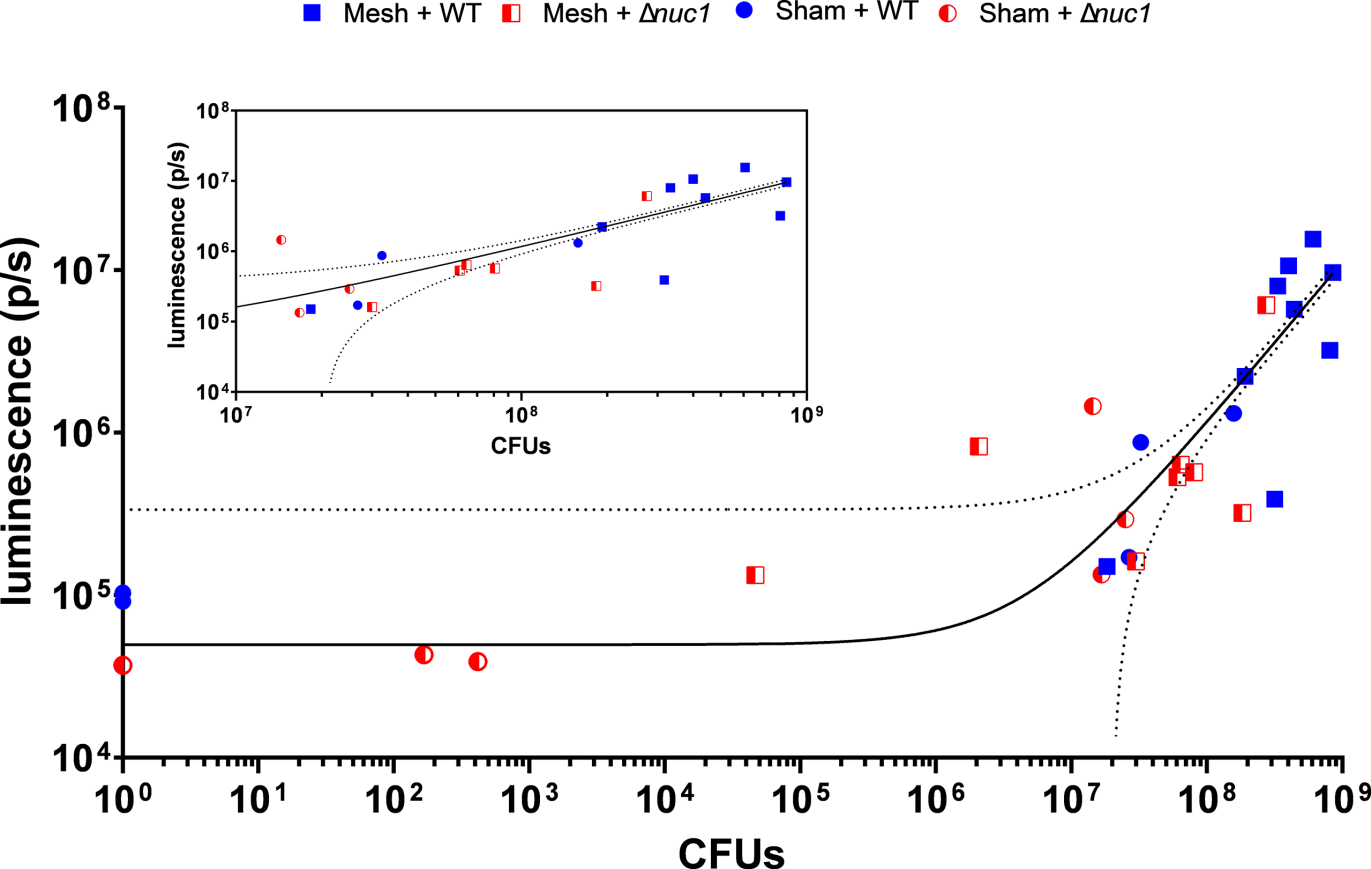
Bacterial bioluminescence as a function of CFU counts of all mice involved in the experiment that were inoculated with either *S. aureus* Newman WT lux or *S. aureus* Newman *Δnuc1* lux. At the inset the same data is shown with an adjusted (cropped) X-axis for more clear inspection of the individual datapoints at the higher end of the X-axis. The black line flanked by dotted line represents a linear correlation with 95% CI, R-squared=0.9395. The lower threshold of the 95% CI intersects the X-axis at a total of 2.1*10^7^ CFUs, indicating the lowest number of bacteria theoretically detectable with using this linear correlation.

Immunohistochemistry was used to identify myeloperoxidase and citrullinated histones which are major components of neutrophil extracellular traps (de Buhr and von Köckritz-Blickwede, 2016). In the *ex-vivo* samples taken from mice with an implant and inoculated with *S. aureus* Newman WT lux, myeloperoxidase (green arrowheads) was located within biofilms at the mesh-tissue interface both 7- and 13 days post implantation (**Figures 3A**, **4A**, compare image 2 with 3). At day 7, mice inoculated with *S. aureus* Newman Δ*nuc1* lux showed diffuse, positive myeloperoxidase staining which was colocalized with positive protein A staining (compare **Figure 3A-7** with **Figure 3A-8**). However, in mice terminated at day 12 and 13, myeloperoxidase was detected mainly in the nuclei of tissue cells in the vicinity of the mesh filament (compare **Figure 4A-7** with **Figure 4A-8**). In the group inoculated with PBS, myeloperoxidase was released (**Figure 3A-13**) at day 7 but accumulated at the mesh-tissue interface at day 13 (compare **Figure 3A-13** with **Figure 4A-13**). Semi quantitative analysis revealed that at day 7, the myeloperoxidase in the group of mice that were inoculated with *S. aureus* Newman WT lux was significantly higher than that in mice inoculated with the *S. aureus* Newman Δ*nuc1* lux (P≤ 0.001) and the group inoculated with PBS (P≤ 0.0001) (**Figure 3C**). Also, at day 13, positive myeloperoxidase staining was detected in the group with the wildtype strain and colocalized with detected protein A but was not significantly higher than in the group inoculated *S. aureus* Newman Δ*nuc1* lux or PBS. In samples taken at day 7, citrullinated histones (orange arrowheads) mainly accumulated at the edge of the biofilms formed by the *S. aureus* Newman WT lux (**Figure 3A-4**). However, in mice terminated at day 12 and 13, citrullinated histones could be found at the interior of biofilm formed by the wildtype strain (**Figure 4A-4**). In samples taken at day 7 from mice inoculated with *S. aureus* Newman Δ*nuc1* lux, citrullinated histones colocalized with positive myeloperoxidase as well in cell nuclei (**Figure 3A-9**) while in mice terminated at day 12 and 13, citrullinated histones mainly colocalized with the nuclei of tissue cells (**Figure 4A-9**). In the group inoculated with PBS, citrullinated histones were found in the nuclei of neutrophils at day 7 but at day 13, citrullinated histones were diffused with slight accumulation at the mesh tissue interface (compare **Figures 3A-14**, **4A-14**). Negative controls with only secondary antibody did not show the characteristic brown DAB precipitate (images 5, 10, 15 of **Figures 3A**, **4A**). Detected citrullinated histones in the group of mice that were inoculated with *S. aureus* Newman WT lux was significantly higher than that in mice inoculated with the *S. aureus* Δ*nuc1* lux at day 7 but not in mice terminated at day 12 and 13 due to a slight increase of citrullinated histones in tissues with the mutant strain (**Figures 3D**, **4D**).

## 4 Discussion

In this study, we analyzed the role of secreted micrococcal nuclease (Nuc1) on *S. aureus* implant infection persistence and biofilm formation in a mouse subcutaneous implantation model involving polyvinylidene-fluoride mesh implants model. A major outcome of this study is that *nuc1* expression facilitates the survival of S*. aureus* Newman WT lux *in-vivo* for at least 7 days after inoculation (**Figures 2** and **3**). This result was anticipated since *S. aureus* Newman WT lux constitutively expresses *nuc1* as a result of a single point mutation in the *SaeS* gene of the SaePQRS regulatory system for nucleases (Olson et al., 2013; Delmain et al., 2020). Therefore, continuous Nuc1 activity will result in ongoing cleavage of the neutrophil extracellular traps DNA backbone and eventually disperse the structure of neutrophil extracellular traps, therewith limiting their antibacterial capacity. Also, *S. aureus* Newman WT lux was able to develop biofilms at the filament-tissue interface, as was clearly observed by immunohistochemistry (white arrows, **Figures 3A**, **4A**, compare images 2, 7 and 12). The majority of the detected protein A in the samples from mice inoculated with Δ*nuc1* were likely related to low amounts of bacteria as the staining was diffused with minor accumulation at the interface of the filaments (**Figures 3A-7**, **4A-7**). Apparently, Nuc1 is important for *S. aureus* biomaterial-associated infection to persist in the acute phase of the foreign body reaction and even extends it, which was also indicated by the lack of foreign body giant cells in mice with the inoculated wildtype strain (**Table 1**) corroborating earlier observations in biomaterial-associated infection studies (van Putten et al., 2011; Sheikh et al., 2015). A recent study also showed that Nuc1 activity contributed to *S. aureus* survival in a hematogenous implant-associated infection model even though Nuc1 had no influence on the bacterial load in peri implant tissue or adherent bacteria on the implant and only *nuc1* and *nuc2* double mutants impacted bacterial load (Yu et al., 2021). This discrepancy in the effect of Nuc1 on *in-vivo* bacterial survival may be due to differences in utilized clinical strain, inoculum concentration, site of implantation and type of biomaterial.

We already anticipated a less severe infection in mice that were inoculated with *S. aureus* Newman Δ*nuc1* lux as related to mice inoculated with the wild type strain. We also expected that a less severe infection would coincide with a higher amount of non-degraded components of neutrophil extracellular traps. Histological analysis, however, provided indicators of the opposite. Production of neutrophil extracellular traps up to day 13 appeared low in mice with the inoculated *S. aureus* Newman Δ*nuc1*. However, secreted Nuc1 in mice with inoculated *S. aureus* Newman WT lux prolonged a the production of myeloperoxidase in the presence of citrullinated histones up to 13 days after mesh implantation (**Figures 3A, C, D** and **4A, C, D**). Since NET-production and biofilm formation coincided in mice with inoculated *S. aureus* Newman WT lux it is suggested that in the presence of a persistent biofilm NET-production is an ongoing process. This might occur in multiple rounds since neutrophils, more abundant in the late acute phase, release neutrophil extracellular traps within minutes after encountering *S. aureus in-vivo* (Yipp et al., 2012) (**Figures 3A**, **4A**, images 2 and 3). Bacteria may escape from neutrophil extracellular traps but apparently do not stop inducing their production, against which they seem to be further protected by their biofilm mode of growth (**Figures 3A**, **4A**, compare images 2, 3 and 4 with 7, 8 and 9). Taken together, Nuc1 production stimulates in *in-vivo* biofilm formation and persistence, rather than inhibit it, as is often found in *in-vitro* assays (Kiedrowski et al., 2011; Tang et al., 2011). This conclusion may not only pertain to *S. aureus* Newman strains with the SaeS^P^ mutation, since also other uncharacterized strains possess this mutation (Cue et al., 2015). The expression of nuclease in *S. aureus* Newman is indeed higher than other *S. aureus* in the first stage of biofilm formation, but nuclease production rises in non-Newman subspecies as well during the later growth phase. Furthermore, nuclease production in clinical isolates is quite variable, and not much lower than in *S. aureus* Newman strains (Rosman et al., 2018).

The hypoxic microenvironment of biofilms may provide protection from neutrophil extracellular traps (Lone et al., 2015; Lone et al., 2015; Wu et al., 2018; Wu et al., 2018). This is because the low concentrations of H_2_O_2_ prevents myeloperoxidase from producing hypochlorous acid (HOCl) (Saed et al., 2009; Davies, 2021), which is a very potent antimicrobial (Parker et al., 2012; Parker et al., 2012). Also, citrullination of histones potentially decreases the antibacterial capacity of histones and exacerbates proteolytic degradation by neutrophil elastase (Tanner et al., 2021) and possibly by biofilm proteases. This proteolytic degradation may explain the low amount of citrullinated histones within the biofilms established at day 7, mainly found at the interface of the filaments in the deeper part of the biofilm where the bacterial activity is lowest (**Figure 3A-4**). However, at day 13, citrullinated histones were found at the interior of the biofilms (compare **Figures 3A-4**, **4A-4**), possibly enabled by metabolically quiescent bacteria with limited production of proteases. The increasing accumulation of citrullinated histones in the interior sections of the biofilms may have contributed to the decrease in the infection of the *S. aureus* Newman WT lux strain at day 13 (**Figures 1**, **2** and **4B**) as citrullinated histones still possess some anti-biofilm activity (Rose-Martel et al., 2017; Arnhold, 2020).

Another reason why biofilms of *S. aureus* Newman WT lux may show resistance against the immune system is the high bacterial density in the biofilm formed, therewith inhibiting easy access to neutrophils and neutrophil extracellular traps. An earlier *in-vitro* study identified that the biofilm formed by Nuc1 producing *S. aureus* Newman WT lux were more compact and the bacterial density (0.5 ± 0.2 bacteria/μm^3^) on a hydrophilic surface was significantly higher than that of Δ*nuc1* (0.3 ± 0.1 bacteria/μm^3^) (Forson et al., 2020). Connective tissue is highly hydrophilic (Warrier et al., 2007; Halper and Kjaer, 2014) and, whereas the PVDF mesh is hydrophobic with a water contact angle above 120 degrees (Boubakri et al., 2015; Kamaz et al., 2019), host serum proteins adsorb onto the mesh *via* hydrophobic interactions, exposing their hydrophilic segments to the surrounding aqueous milieu (Hirsh et al., 2013). This difference in biofilm density may imply that neutrophil entry and diffusion of neutrophil extracellular trap components into biofilms formed by the *S. aureus* Newman *Δnuc1* bacteria is facilitated.

While neutrophil extracellular traps-production seemed to be an ongoing process, degradation of the DNA backbone of these traps by Nuc1 in mice with the inoculated WT strain persisted as well. A strong, indirect evidence of neutrophil extracellular traps and neutrophil extracellular trap-DNA-degradation by Nuc1 in this study, is the lack of immune cells (including macrophages) in the vicinity of the mesh at day 7 and 13 (**Figures 3A** and **4A**, compare images 1 with 6, 11). This observation may be due to induced apoptosis of immune cells by the conversion of mono- and di-nucleotides into deoxyadenosine by staphylococcal adenosine synthase A, a 3’- and 5’ nucleotidase. Once deoxyadenosine is transported into the cytosol of macrophages, it can induce caspase 3-mediated cell death (Thammavongsa et al., 2013; Winstel et al., 2018). Also, leukocidins are capable of lysing neutrophils (Geiger et al., 2008; Collins et al., 2020). However, leukocidins produced in the absence of Nuc1 did not result in the death of neutrophils at day 7 (compare **Figures 3A-1** and **3A-6**). This finding suggests that Nuc1 may also play a role in a leukocidin-mediated mechanism that results in the apoptosis of neutrophils as was suggested earlier (Bhattacharya et al., 2020). At day 13, more immune cells were able to survive in close proximity to the filaments and in the presence of the WT bacteria, potentially due to a reduced Nuc1 expression by a more metabolically quiescent biofilm (compare **Figure 3A-1** with **Figure 4A-1**).

The presently applied murine model is well established for preclinical infection studies allowing real time monitoring of the bioluminescent biofilm formation in one and the same mouse during the course of the infection (Engelsman et al., 2009; Daghighi et al., 2012). Starting at 9 days post-implantation/infection, some mice with an implanted mesh and an infection (both bioluminescent *S. aureus* Newman WT lux and Δ*nuc1* strains), started to develop such an efflorescent infection that their implant was rejected or became visible, which met humane endpoints defined for this study. This was a limiting factor in our follow-up period, potentially due to the virulent character of the *S. aureus* Newman. Comparatively, *S. aureus* Xen36, another luminescent strain, allowed mesh implants to remain in place during 25 days while adhering to these same humane endpoints (Daghighi et al., 2012). By comparing the number of CFUs on the implant with the bioluminescence measured before termination, we found a linear correlation (R^2 =^ 0.93) between CFUs and luminescence. However, there is a bioluminescence detection limit of about 2*10^5^ p/s, where the luminescent signal only increased above this limit at bacterial loads exceeding 10^7^ CFU (**Figure 5**). This coincides with earlier found values with regard to sensitivity of bioluminescence measurements (Sjollema et al., 2010).

## 5 Conclusion

Our results suggest that *S. aureus* Nuc1 production stimulates biofilm formation and infection persistence in a mouse subcutaneous implant model. Nuc1 has a persistent effect on the local host immune response resulting in dissolution of NETs, allowing bacteria to survive, inducing ongoing NET release, and causing a lack of immune cells potentially by cell apoptosis. Nuc1 producing *S. aureus* formed biofilms on implants and in surrounding tissues that were persistent for at least 12 days after implant infection. Specifically targeting micrococcal nuclease production could be a novel strategy in preventing *S. aureus* BAI.

## Data Availability Statement

The raw data supporting the conclusions of this article will be made available by the authors, without undue reservation.

## Ethics Statement

The animal study was reviewed and approved by the Competent Authority (Centrale Commissie Dierproeven, CCD, Den Haag, The Netherlands) (IvD protocol 197305-01-001).

## Author Contributions

CR contribute to conceptualization of the study, methodology animal experiment and *ex vivo*, formal analysis animal experiment and *ex vivo*, investigation animal experiment and *ex vivo*, resources, data curation, writing—original draft, writing—review and editing, visualization animal experiment and *ex vivo*. AF contributed to conceptualization of the study, methodology histology, formal analysis histology, investigation histology, writing—original draft, writing—review and editing, visualization histology. TK contributed to methodology animal experiment and ex-vivo, validation animal experiment, resources, writing—review and editing. HM contributed to writing—review and editing, supervision, funding acquisition. JS contributed to conceptualization of the study, methodology, resources, writing—review and editing, supervision, project administration, funding acquisition. All authors approved the manuscript and are informed of this submission.

## Funding

AF acknowledges the financial support of the European Union’s Horizon 2020 research and innovation program under the Marie Skłodowska-Curie grant agreement 713482 (ALERT Cofund).

## Conflict of Interest

The authors declare that the research was conducted in the absence of any commercial or financial relationships that could be construed as a potential conflict of interest.

## Publisher’s Note

All claims expressed in this article are solely those of the authors and do not necessarily represent those of their affiliated organizations, or those of the publisher, the editors and the reviewers. Any product that may be evaluated in this article, or claim that may be made by its manufacturer, is not guaranteed or endorsed by the publisher.
