# Supplementary material for: Micrococcal Nuclease stimulates Staphylococcus aureus Biofilm Formation in a Murine Implant Infection Model
Source: Front Cell Infect Microbiol. 2022 Jan 17;11:799845. doi: 10.3389/fcimb.2021.799845 (PMC8801922; doi:10.3389/fcimb.2021.799845)
Supplement: Supplementary file 1 [file DataSheet_1.docx]

***Supplementary Material***

to

**Micrococcal nuclease is essential for *Staphylococcus aureus* biofilm formation in a murine implant infection model**

**Abigail M. Forson, Colin W.K. Rosman, Theo G. Van Kooten, Henny C. van der Mei, Jelmer Sjollema**

University of Groningen, University Medical Center Groningen, Department of Biomedical Engineering, A. Deusinglaan 1, 9713 AV, Groningen, The Netherlands

**Table S1.** Animal groups**.**

|  | ***7 days*** | | | | | |
| --- | --- | --- | --- | --- | --- | --- |
|  | ***with mesh*** | | | ***without mesh*** | | |
| ***Group #*** | ***S. aureus Newman,* Δnuc1 lux*,*** | ***S.aureus Newman* lux** | ***Sterile PBS*** | ***S. aureus* Newman Δ*nuc1* lux,** | ***S.aureus* Newman lux** | ***sterile PBS*** |
| 1 | 7 |  |  |  |  |  |
| 2 |  | 7 |  |  |  |  |
| 3 |  |  | 4 |  |  |  |
| 4 |  |  |  | 3 |  |  |
| 5 |  |  |  |  | 3 |  |
| 6 |  |  |  |  |  | 3 |
|  | ***13 days*** | | | | | |
|  | ***with mesh*** | | | ***without mesh*** | | |
|  | ***S. aureus Newman,* Δnuc1 lux*,*** | ***S.aureus Newman* lux** | ***Sterile PBS*** | ***S. aureus* Newman Δ*nuc1* lux,** | ***S.aureus* Newman lux** | ***sterile PBS*** |
| 7 | 3 |  |  |  |  |  |
| 8 |  | 3 |  |  |  |  |
| 9 |  |  | 3 |  |  |  |
| 10 |  |  |  | 3 |  |  |
| 11 |  |  |  |  | 3 |  |


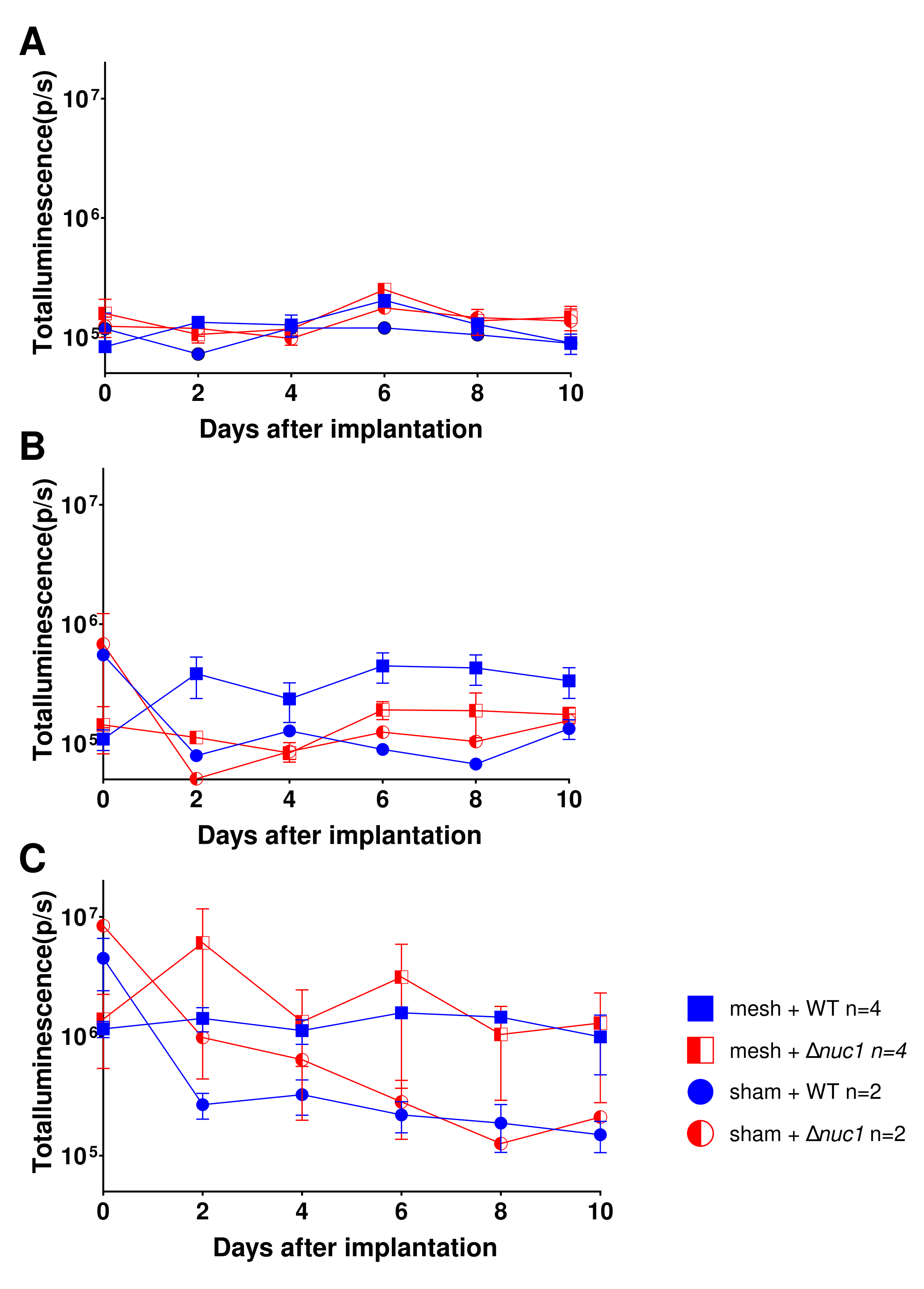


**Figure S1.** Dose finding study in 6 groups of mice inoculated with A) 10^6^ CFU, B) 10^7^ CFU, or C) 10^8^ CFU of *S. aureus* Newman WT lux (WT) or a nuc1 deficient strain (Δ*nuc1*). At T=0 days all mice were implanted with a mesh or received a sham surgery prior to infection. Bioluminescence radiance measurements were made at several time points during the course of infection. Differences between luminescence in mice with and without implants were only obtained when mice were inoculated with 10^8^ CFU. Results of these measurements (n=4) did not significantly differ from the main experiments (n=10).

**Figure S2**. Survival of mice with mesh implants and inoculated with either *S. aureus* Newman WT lux (WT), *S. aureus* Newman *Δnuc1* lux (*Δnuc1*), or sterile PBS. Survival in all sham surgery groups was 100%; these groups are not included in this graph. Animal death, loss of implant, or reaching any humane endpoint, were treated as valid event. Censored events (vertical ticks) were planned animal termination at T=7 and 13. Median survival of both mesh + WT and mesh + Δ*nuc1* groups is 12 days.


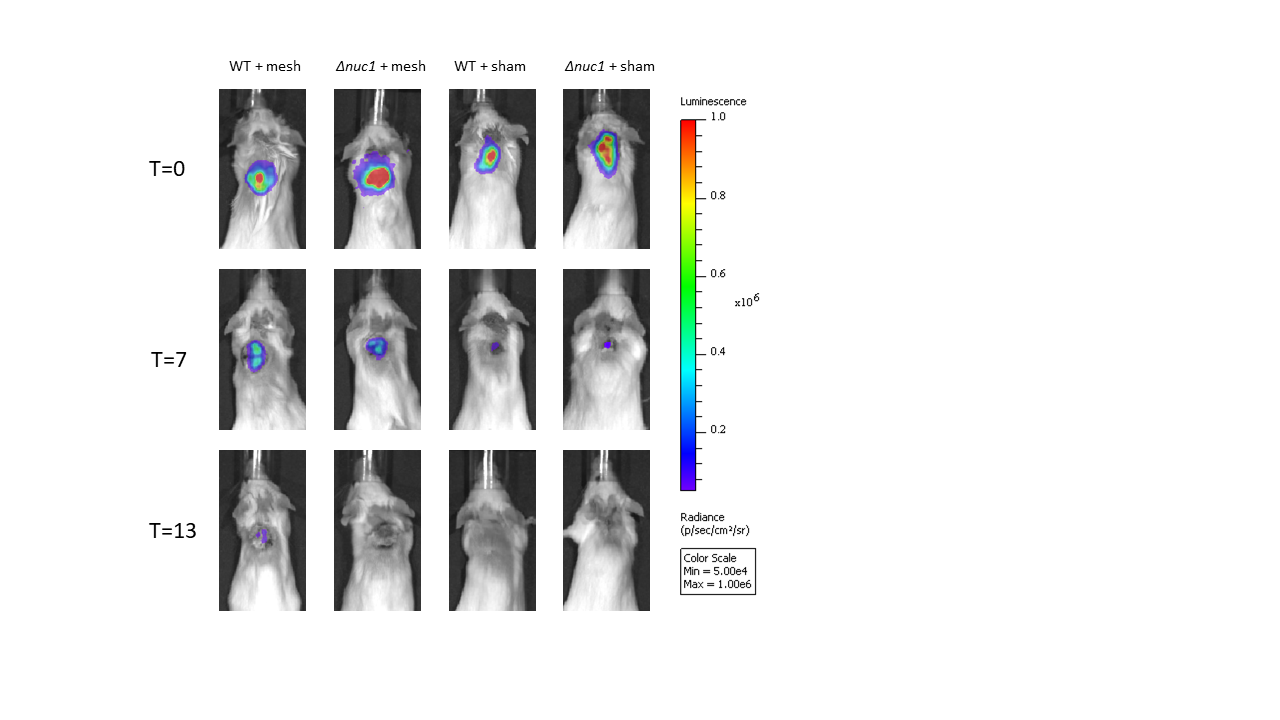


**Figure S3**. Typical image overlays of mouse images with luminescence radiance intensities indicated by color as validated in the radiance scale on the right. Bioluminescence radiance is indicated in p/s/cm2/str. Mice from 4 groups are included as observed at 3 different time points during the course of infection. Two groups obtained a subcutaneously implanted mesh and 2 groups only obtained a subcutaneous pocket (sham). Both groups of mice were inoculated with either S. aureus Newman lux (WT) or with a nuclease deficient mutant (*Δnuc1*).


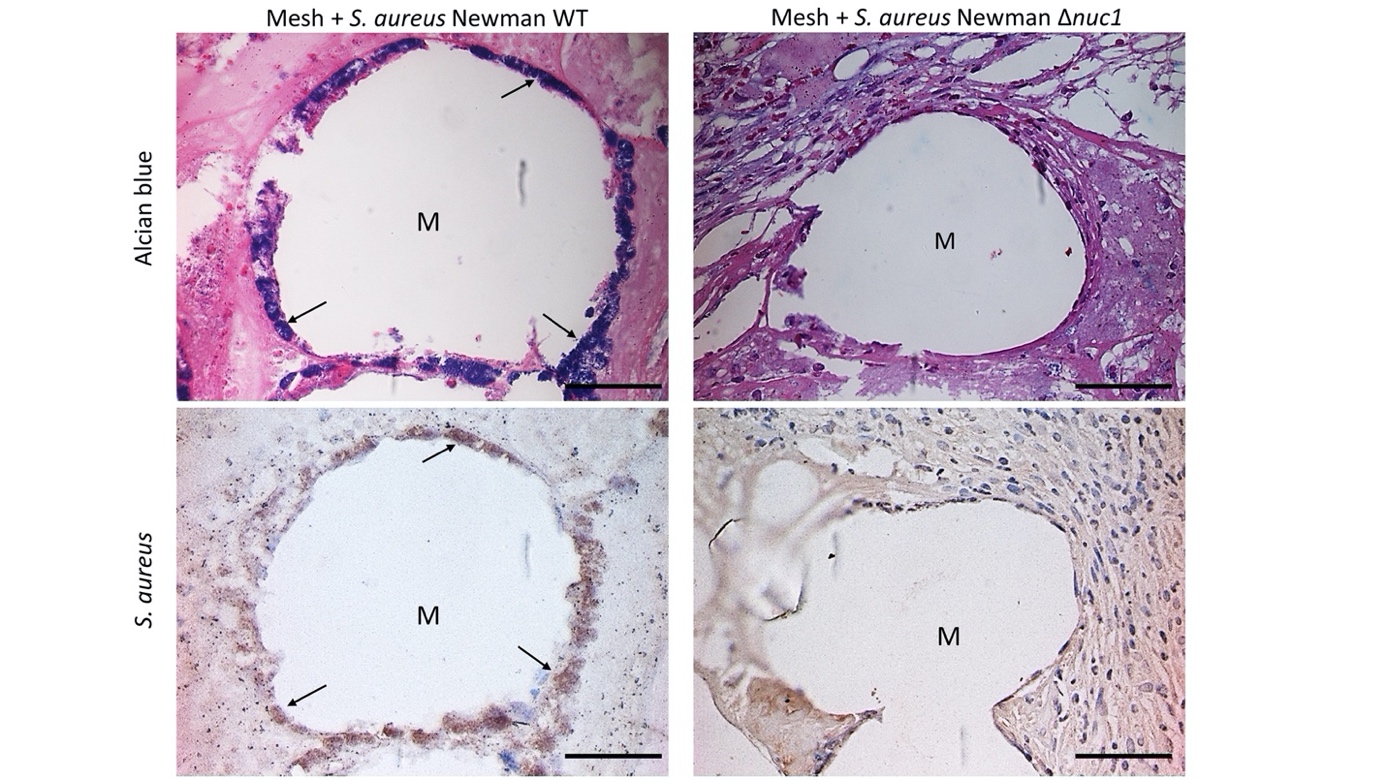


**Figure S4.** Representative images of (upper row) eosin (pink) staining with alcian blue and (lower row) hematoxylin (dark blue), eosin (pink) staining and immune-histochemical detection (brown staining) of *S. aureus* in mice tissue biopsies at positions surrounding a single filament of a PVDF surgical mesh implant (M) in combination with an *S. aureus* Newman WT lux or *S. aureus* Newman Δnuc1 lux, 7 days after implantation.
